# Early detection of antiangiogenic treatment responses in a mouse xenograft tumor model using quantitative perfusion MRI

**DOI:** 10.1002/cam4.177

**Published:** 2014-01-06

**Authors:** Reshmi Rajendran, Wei Huang, Annie Mei Yee Tang, Jie Ming Liang, Stephanie Choo, Torsten Reese, Hannes Hentze, Susan Boxtel, Adam Cliffe, Keith Rogers, Brian Henry, Kai Hsiang Chuang

**Affiliations:** 1 MRI Group, Singapore Bioimaging Consortium, Agency for Science, Technology and ResearchSingapore; 2 Translational Medicine Research Centre, MSDSingapore; 3 Institute of Molecular and Cellular Biology, Agency for Science, Technology and ResearchSingapore; 4 Clinical Imaging Research Centre, National University of SingaporeSingapore; 5 Department of Physiology, Yong Loo Lin School of Medicine, National University of SingaporeSingapore

**Keywords:** Angiogenesis, arterial spin labeling, blood flow, dynamic-contrast enhanced MRI, magnetic resonance imaging, tumor, xenograft

## Abstract

Angiogenesis plays a major role in tumor growth and metastasis, with tumor perfusion regarded as a marker for angiogenesis. To evaluate antiangiogenic treatment response in vivo, we investigated arterial spin labeling (ASL) magnetic resonance imaging (MRI) to measure tumor perfusion quantitatively. Chronic and 24-h acute treatment responses to bevacizumab were assessed by ASL and dynamic-contrast-enhanced (DCE) MRI in the A498 xenograft mouse model. After the MRI, tumor vasculature was assessed by CD34 staining. After 39 days of chronic treatment, tumor perfusion decreased to 44.8 ± 16.1 mL/100 g/min (*P* < 0.05), compared to 92.6 ± 42.9 mL/100 g/min in the control group. In the acute treatment study, tumor perfusion in the treated group decreased from 107.2 ± 32.7 to 73.7 ± 27.8 mL/100 g/min (*P* < 0.01; two-way analysis of variance), as well as compared with control group post dosing. A significant reduction in vessel density and vessel size was observed after the chronic treatment, while only vessel size was reduced 24 h after acute treatment. The tumor perfusion correlated with vessel size (*r* = 0.66; *P* < 0.005) after chronic, but not after acute treatment. The results from DCE-MRI also detected a significant change between treated and control groups in both chronic and acute treatment studies, but not between 0 and 24 h in the acute treatment group. These results indicate that tumor perfusion measured by MRI can detect early vascular responses to antiangiogenic treatment. With its noninvasive and quantitative nature, ASL MRI would be valuable for longitudinal assessment of tumor perfusion and in translation from animal models to human.

## Introduction

Angiogenesis, the process by which new blood vessels are formed to supply oxygen and nutrients to cells, is known to be instrumental for tumor growth and metastasis. Antiangiogenic drugs like bevacizumab, sorafenib 1, axitinib 2,3, and pazopanib 4 are either approved as first-line treatment or being used in clinical trials either alone or in combination 5 to interfere with angiogenic ligands, their receptors or downstream signaling, to upregulate or deliver endogenous inhibitors, or to directly target tumor vasculature 6. Traditionally, angiogenesis has been evaluated by the microvascular density (MVD) in histology, but this invasive methodology suffers from sampling bias. Various imaging techniques like ultrasound, computed tomography (CT), positron emission tomography and magnetic resonance imaging (MRI) have been applied preclinically and clinically to image angiogenesis in oncology and assess response to therapy longitudinally 7–12.

MRI assessment of tumor angiogenesis has been carried out most commonly by dynamic-contrast-enhanced (DCE) imaging of Gd-chelates 13,14 and, in very few instances, by arterial spin labeling (ASL) of tissue perfusion 15. DCE-MRI has been widely used in preclinical and clinical trials of antiangiogenic treatments. Preclinically, the most common applications of DCE-MRI has been to either understand the characteristics of angiogenesis 16, evaluate or compare the efficacy of therapy 17, or to study changes in perfusion as a downstream effect of angiogenesis 18. Bevacizumab, the first vascular endothelial growth factor (VEGF)-targeting drug officially approved for cancer therapy is a humanized monoclonal antibody IgG1 that blocks the binding of human VEGF to its receptors, thus disrupting autocrine and paracrine survival mechanisms mediated by VEGF-1 and VEGF-2 19. Antiangiogenic effects of bevacizumab and other drugs like Cediranib 17, Vandetanib 20 Sunitinib 21, or compounds like liposomal prednisolone phosphate 22 have been examined with DCE-MRI. Furthermore, DCE-MRI has also been applied in detecting early responses to treatments. In one study, colon tumor xenografts treated with bevacizumab was evaluated as early as 36 h after treatment and tumor growth was shown to correlate with *K*^trans^ and fPV in tumor periphery 23.

Although DCE-MRI has been the most widely used method in monitoring tumor response to therapy, it has certain limitations. Major concerns are the models used for quantification, the arterial input function, and the estimation of Gd concentration. For example, the transfer coefficient, *K*^trans^, often correlated with angiogenesis, is influenced by both blood flow and permeability; hence, it is difficult to determine whether the change is due to flow or permeability. Another discrepancy that is often overlooked is the fact that, while the contrast medium is confined to the extracellular space, the bulk of tissue water is intracellular. Hence, transmembrane water exchange can affect the accuracy of the calculated tissue contrast agent concentration 24. Besides the ambiguity with modeling parameters for quantification, another limitation with DCE is the known side effect of the development of nephrogenic systemic fibrosis, which limits application to patients without renal impairments, to allow a rapid clearance of the contrast agent from the body.

ASL, on the other hand, is a noninvasive and quantitative technique that measures perfusion by magnetically labeling water as a freely diffusible endogenous tracer. Several studies have used ASL to measure perfusion in brain tumor in humans 25–29, and in rodents 30,31. However, reports using ASL in mouse tumor outside the brain have been limited. Besides artifacts caused by movement, susceptibility difference, magnetic field inhomogeneity and fat near the abdomen, the sensitivity of ASL to low perfusion in tumor makes imaging especially difficult. A study compared ASL and DCE-MRI in mouse tumor and found high correlation between blood flow and K_ep_, the rate constant of transfer between the extracellular space and the blood plasma 32. However, large variability and very high flow (207 ± 111 mL/100 g/min) values were reported in center of tumor. The other study compared ASL in three tumor models in a longitudinal study of Sorafenib treatment and demonstrated tumor blood flow from 10 to 100 mL/100 g/min with significant change by the treatment seen after 3 days 33. The follow-up study showed that tumor perfusion measured by ASL correlated with MVD and the perfusion changes reflected different antiangiogenic treatment design 34.

In all these studies, treatment responses over days or months were reported, but it has not been shown whether perfusion can reflect response at the earliest time, such as in 24 h. In this study, we investigated both DCE-MRI and ASL perfusion imaging for detection of chronic as well as 24 h acute responses to antiangiogenic treatment with bevacizumab. Using a previously optimized ASL sequence 35, low perfusion can be reliably measured and quantified. Perfusion in the tumor showed significant change compared to isotype control in the chronic treatment and to pretreatment in the 24 h treatment study. The finding was further validated with CD34 vessel staining in histology.

## Materials and Methods

### Animal preparation

The animal study was approved by the local Institutional Animal Care and Use Committee (Biomedical Sciences Institute, Agency for Science Technology and Research, Singapore). The guidelines that were followed for acceptable toxicity for the drug were as defined by the National Cancer Institute (NCI) as mean group body weight loss less than 20% during the study; none of the enrolled animals showed treatment-related toxicity.

The A498 cell line (ATCC, Manassas, VA), a renal carcinoma, was cultured in standard cell culture medium, containing advanced minimum essential medium (advanced MEM; Gibco, Carlsbad, CA) supplemented with 10% heat-inactivated fetal calf serum (FCS; Hyclone, Logan, UT), 2 mmol/L l-glutamine and 1% penicillin/streptomycin (S/P, Gibco) to sufficient amount for inoculation. We confirmed the VEGF-producing capability of the cells by enzyme-linked immunosorbent assay (ELISA) (results not shown). Tumors were induced in 8-week-old female anaesthetized BALB/cOlaHsd-*Foxn1*^*nu*^ nude mice (mixture of 2–3% isoflurane in O_2_ at a flow of 0.6 L/min) by inoculating A498 cells suspended in 0.1 mL of 50% Matrigel™ (BD Biosciences, San Jose, CA) in Dulbecco's phosphate-buffered saline (DPBS; Gibco) intradermally at the right flank of each mouse. The tumor growth was continuously monitored by measuring the tumor width and length 2–3 times per week with a caliper, where the length being defined as the longest side and the width as the perpendicular to the length. The measurement was carried out by a single operator over the whole study period for consistency. The tumor volume (mm^3^) was calculated by the following equation:


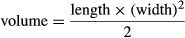
(1)

### Experimental design

The sensitivity of our quantification technique to changes in intratumor perfusion was assessed by comparing control animals treated with isotype antibody to animals treated with the anti-human VEGF drug bevacizumab (Avastin®, Roche, Indianapolis, IN), which would potentially inhibit tumor growth and impact on tumor perfusion. Two different study designs were used.

**Figure 1:**
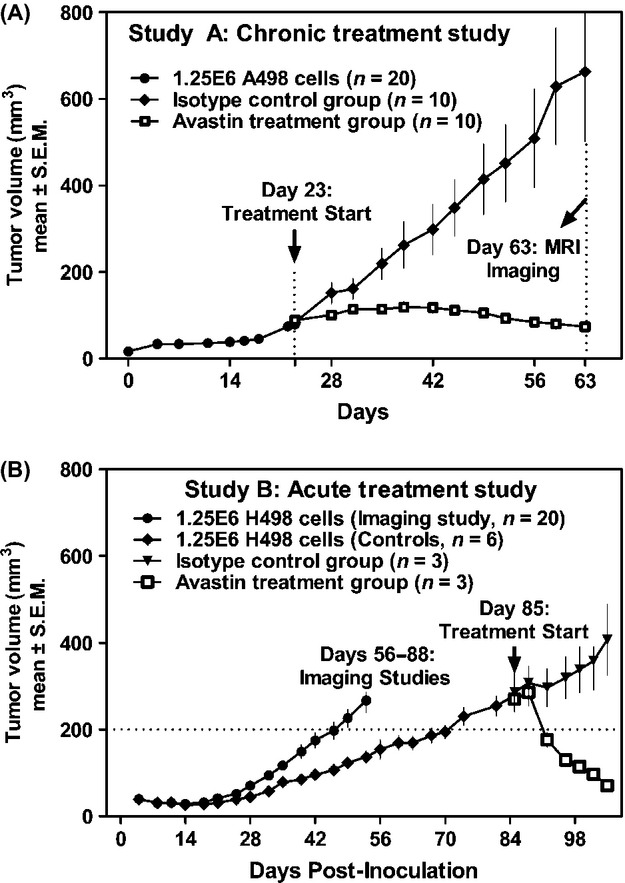
Tumor growth curves for chronic and acute treatment studies. BALB/cOlaHsd-Foxn1nu nude mice were inoculated with 1.25 million A498 cells intradermally, and tumor volumes were determined by standard caliper measurements. Group means and SEM are shown. Tumor growth curves for the chronic treatment study (A). Bevacizumab was used at a dose of 5 mg/kg i.p. twice per week (Monday and Thursday), and human IgG1 Kappa isotype control was given with the same regimen. Tumor growth curve for all animals that went into the acute treatment study (B); between days 56 and 88 post inoculation, animals were either treated with bevacizumab or with isotype control antibody once, and imaged the following day (24 h later). Six separate control animals from the same tumor inoculation were randomized into two groups at day 85, and then treated with bevacizumab or with isotype control antibody for 20 days with the same twice per week regimen as used in the chronic treatment study.

For the chronic treatment study, initially 40 animals were inoculated with 1.25 million A498 cells, and once the tumor volume reached around 80 mm^3^ at day 23 post inoculation, 20 animals were selected for the study, and then randomly divided into two groups with equal tumor volumes (*n* = 10/group, one animal per group was later excluded from imaging for technical reasons). As for the rest of the 20 animals, we deselected 10 animals due to absence or too rapid tumor growth, and used a group of 10 animals for vehicle treatment. These animals showed an indistinguishable growth from the isotype group shown in Figure 1A. Animals were treated either with the monoclonal anti-VEGF bevacizumab (5 mg/kg i.p., twice per week), or an equal amount of a human IgG1 Kappa isotype control antibody (The Binding Site, Birmingham, UK). To obtain dosing solutions, the 25 mg/mL stock solution of Avastin was diluted in sterile PBS to obtain 0.5 mg/mL for an injection volume of 10 mL/kg body weight. For the isotype control, the stock concentration of 5 mg/mL was diluted to 0.5 mg/mL in sterile PBS to administer the mice at a dosage of 5 mg/kg body weight i.p. Antibody solutions were prepared fresh daily in an autoclaved glass vial and capped to close in a biosafety cabinet. All tumors were imaged at day 63, 63 days post inoculation (Fig. 1A). Due to scan time limitations, these experiments were staggered so that strict adherence to time points was possible.

For the acute treatment study on larger tumors, 30 animals were inoculated with A498 cells. When individual tumors reached a tumor volume above 200 mm^3^, the 20 selected animals were subjected to acute treatment with 5 mg/kg i.p. bevacizumab or human IgG1 Kappa isotype control antibody (Fig. 1B), and this time were imaged pre- and 24 h post treatment. Selected animals were treated between days 56 and 88 (for details see Fig. 1B), and the average tumor volumes of all treated tumors was 443 ± 119 mm^3^. Additionally, we selected a small subset of animals to serve as treatment control for larger tumors to demonstrate that bevacizumab treatment is efficacious in this experimental situation. When tumors reached a volume of 270 mm^3^ (at day 85, Fig. 1B), these animals were treated with the same dose of 5 mg/kg i.p. bevacizumab or human IgG1 Kappa isotype control antibody twice per week for 20 days.

For MRI studies, mice were anesthetized with a mixture of 3% isoflurane in air and O_2_ (approximately 2:1) for induction, and reduced to 1–1.5% for maintenance in MRI by a nose cone to maintain regular breathing at a frequency of 80 ± 10 breaths/min monitored by an MRI-compatible sensor (SA Instrument Inc., Stony Brook, NY). Respiration and body temperature of the animals were monitored throughout the scan. The temperature, measured rectally, was maintained by an air heater at 37°C.

### MRI parameters

The experiments were carried out on a 7T MRI (ClinScan, Bruker Biospin GmBH, Ettlingen, Germany) with a 20-cm bore size and a high-performance gradient and shim coil (gradient strength of 630 mT/m, slew rate of 6300 T/m per second) interfaced to a Siemens (Erlangen, Germany) console. A volume coil (diameter: 72 mm) was used for RF transmit. To improve sensitivity of ASL, a 10-mm receive surface coil was placed close to the region of interest.

Anatomical turbo spin echo (TSE) *T*_2_-weighted images covering the whole tumor were acquired on 14 slices of 1-mm thickness without any gap between them, with a TR of 1500 msec and a TE of 36 msec with two averages. For perfusion imaging using ASL, a single axial slice of 2 mm thickness in a section of the tumor that looked as heterogeneous as possible from the *T*_2_-weighted image and crossing the right kidney if possible (for validation) was acquired. The rationale for choosing a heterogeneous area in the tumor was to capture various characteristics, such as necrotic core and tumor periphery, to understand the diversity of perfusion in the tumors better. The spin labeling was performed using the Flow-sensitive Alternating Inversion Recovery (FAIR) technique 36,37 with interleaving nonselective (global) adiabatic inversion (with a hyperbolic secant pulse) and slice-selective inversion of 5 mm thick, and multiple inversion times of 100, 350, 750, 1500, 2500, and 4000 msec. Spin-echo EPI of TR = 6 sec, TE = 18 msec, field of view (FOV) = 28 × 21 mm^2^, and resolution = 0.44 × 0.44 mm^2^ and 40 measurements were used to acquire the ASL images (one average, matrix size = 64 × 48, total imaging time = 24.36 min, slice thickness = 2 mm, and slice-selective inversion = 5 mm). For perfusion quantification, a separate *T*_1_ map was acquired using inversion recovery and the same spin-echo EPI acquisition with TR = 10 sec and TI = 10, 100, 500, 1000, 2000, 5000, and 8000 msec (two averages and total imaging time of 4 min). The EPI data were reconstructed with zero-filling to obtain same in-plane resolution as the DCE data.

For DCE-MRI, a time series of 3D FLASH sequence with the following parameters was used: TR/TE = 4.25/1.47 msec, flip angle = 6°, FOV = 28 × 16.8 mm^2^, matrix = 128 × 76, in-plane resolution = 0.218 × 0.218 mm^2^, slice thickness = 1 mm, number of phase encoding steps in the slice direction = 8, number of dynamics = 100, and a temporal resolution of 3.1 sec per dynamic. To make the slices comparable to ASL, care was taken to ensure that two slices from the 3D volume, which comprised of eight 1 mm slices, acquired for DCE corresponded to the ASL scan. These two slices were later averaged to derive perfusion-related parameters during data processing. Intravenous bolus injection of gadopentetate dimeglumine (Gd-DTPA) was performed at the tail vein at a dose of 0.2 mmol/kg followed by a flush of 0.2 mL of physiological saline, at dynamic 6 after the start of image acquisition. *T*_1_ mapping for DCE analysis was done prior to contrast agent injection, using the same set of imaging parameters, while having variable flip angles ranging from 2° to 20° with 2° of increment and three averaging 38,39.

### Histology

At the end of the last MRI experiment, each mouse was euthanized and the tumor was extracted and prepared in paraffin. The slice location for histology was selected based on the distance of the ASL imaging slice to both ends of the tumor along the *z*-axis measured from the anatomical MRI. Rat anti-CD34 (Cat # Sc-1506; Santa Cruz, Dallas, TX) immunohistochemical staining was conducted to stain for blood vessels. After dewaxing and dehydrating, heat-induced epitope retrieval was performed using Bond™ Epitope Retrieval Solution (Singapore, Singapore) 1 (pH 6) for 40 min at 100°C. Slides were then cooled to room temperature with four washes of 1× Bond™ Wash Solution. Endogenous peroxidase blocking was performed for 30 min at room temperature in 3–4% (v/v) H_2_O_2_, followed by three rinses in 1× Bond™ Wash Solution. Slides were incubated with primary antibody at the appropriate concentration for 60 min. At the end of the incubation, the slides were rinsed three times in 1× Bond™ Wash Solution. Secondary antibody (goat anti-rat HRP, Invitrogen, Carlsbad, CA; 1:50) was added for 30 min and then the slides were rinsed three times in 1× Bond™ Wash Solution. Bond™ Mixed DAB Refine was applied for 5 min, following which the slides were rinsed in deionized water to stop the DAB reaction. Counterstaining with hematoxylin was performed for 5 min. After this, the slides were rinsed in deionized water and 1× Bond™ Wash Solution. Slides were finally dehydrated and mounted in synthetic mounting media and sectioned into 5 *μ*m thick slices.

### Data analysis

#### Quality control

Movement in ASL data was visually inspected and no significant movement was observed in all the animals. Since EPI used in ASL acquisition is sensitive to field inhomogeneity, which could cause distortion in the images and hence biasing the estimation of flow, the degree of distortion was evaluated by comparing with the corresponding FLASH image. Jaccard index (J-index), which measures the degree of overlap of two areas, was used to quantify the severity of distortion, with reference to the precontrast FLASH image acquired at the same location for DCE analysis. ROIs were drawn by two independent operators blinded to the treatment regimen at the boundary of the tumor on both EPI and FLASH images, and the J-index was calculated by the following equation:


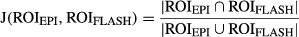
(2)

J-index ranges from 0 to 1. If the two ROIs are exactly completely overlapped, the J-index equals 1, which would indicate that the EPI data have no distortion with reference to the FLASH image.

#### Flow quantification

The ASL magnetization differences between control and label images at different TIs, *ΔM*(TI), was fitted to the following kinetic function 40 by a nonlinear least square routine in Matlab (Mathworks, Nattick, MA) to derive the quantitative perfusion, *f*,


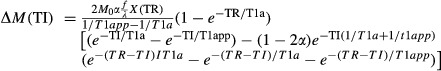
(3)

where 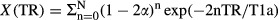, which is a constant with dummy *N* ≥ 4. *M*_0_, the equilibrium magnetization per unit mass of tissue, and *T*_1app_, the apparent longitudinal tissue relaxation time defined as 1/*T*_1app_ = 1/*T*_1_ + *f*/*λ*, were determined from the three parameter fit of the *T*_1_ mapping data. The inversion efficiency, *α*, was also calculated and found to be close to one in sample cases and was therefore assumed to be one for the entire group. Other parameters employed in the calculation were: *λ*, blood/tissue partition coefficient, which was chosen to be 0.9 mL/g based on studies where the blood water content was also adjusted for the density of blood 41 and *T*_1a_, the longitudinal relaxation time of arterial blood, which was assumed to be 2210 msec at 7T 42. The transit time was ignored as the delivery of spins is known to be almost instantaneous with inversion slice thickness as used in this study. In addition, our previous experiments on mouse renal perfusion had indicated the absence of transit time 43.

To determine tumor perfusion, tumor ROI was manually drawn at the boundary of the tumor based on the nonselective inversion image, which showed a clearer tumor boundary than in the ASL perfusion map. Anatomical *T*_2_ and FLASH images at the same slice location were also displayed for references. The ROI was drawn carefully to include the whole tumor, and avoid locations of major arteries, which appeared as bright spots in the FLASH image. The ROI was then overlaid concurrently onto the ASL flow map and coefficient of determination (*R*^2^) map obtained from *T*_1_ mapping. Only values with *R*^2^ > 0.9 were included. From the ASL perfusion map, the mean and the histogram of blood flow within the tumor ROI was calculated. Mean blood flow of kidney cortex was also computed as a reference.

#### DCE-MRI

Semi-quantitative parameters were then evaluated on the DCE-MRI data. Normalized signal intensity versus time curve was calculated on a voxel-by-voxel basis, to reflect the signal intensity changes (*Δ*SI(*t*)) with reference to the precontrast baseline, as follows:


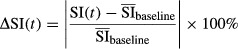
(4)

where SI(*t*) and 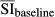 were the signal intensity at time *t* and mean of signal intensity of precontrast baseline, respectively. The bolus arrival time was determined for every experiment, to avoid any inconsistency in bolus injection time due to manual injection. In each voxel, the time of contrast arrival, T0, was defined as the time at which the voxel signal intensity change becomes larger than the following threshold 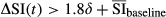, where *δ* is the standard deviation of signal change over the baseline period. The earliest arrival time among all voxels was regarded as the actual bolus arrival time. The voxel-wise time of maximum contrast enhancement (*T*_max_) map, area under normalized signal intensity time curve (AUC) maps integrated for the first 60, 90, 150 sec, and *T*_max_ after the bolus arrival were computed. Again, ROI was manually drawn at the boundary of the tumor on the precontrast baseline image, and overlaid onto the T0, *T*_max_ and AUC maps for calculating the respective mean values within the tumor ROI. The AUC of first 60 sec (AUC60) was found to be more sensitive and was used in the following comparison.

#### Histology analysis

For analysis of histological sections, slides were scanned using an Ariol SL-50 slide scanner and 20× objective (Leica Microsystems, Wetzlar, Germany). Analysis was performed using Ariol software (Leica Microsystems). Histological sections were aligned using the Ariol SlideLink function. This allows the precise alignment of sections based on shape and structural landmarks. Anatomical MRI images were compared to histological sections and matched based on overall section shape and other obvious landmarks. Tumors where anatomical MRI images and histological sections could not be unambiguously aligned were excluded from further analysis. CD34 staining was quantified using the AngioSight algorithm, to measure microvessel size and distribution. Results were correlated with corresponding regions in the MRI scans.

#### Statistical analysis

The ROIs corresponding to the whole tumor were compared between the control and the treated groups, and between ASL and DCE. Pearson's correlation was calculated between the ASL and DCE. Two-way analysis of variance (ANOVA) and Student *t*-test were used to evaluate the statistical significance and *P* < 0.05 regarded as significant.

## Results

### Chronic treatment study (Study A)

The animal model of choice was the angiogenesis-driven, slow-growing human renal cancer cell xenograft A498 model. A498 cells produce large amounts of VEGF, and therefore this model was previously found most suitable for the investigation of anti-VEGF/antiangiogenesis treatment mechanisms 44,45. The A498 xenograft tumor model was established in pilot studies using various amounts of inoculated cells. For following experiments used for MRI studies, we inoculated mice with 1.25 million cells. We consistently observed a linear growth curve as described previously 45–47 starting 20–25 days after intradermal cell inoculation.

For the chronic bevacizumab treatment study (Study A), we inoculated initially 40 animals, from which 20 animals were selected for the study groups shown in Figure 1A. The mean tumor volume was 86 mm^3^ at day 23 post inoculation when the bevacizumab and isotype control antibody treatments started. The bevacizumab treatment lasted until day 63, when the tumors of all animals were assessed by MRI, followed by necropsy and histological analysis of all tumors.

At day 63, the mean tumor volume in the isotype-treated group had increased to 663 mm^3^, whereas tumor volumes in the bevacizumab-treated group showed, after an initial slight growth phase, an overall slight decline in tumor volume to 74 mm^3^ (Fig. 1A). This difference in tumor growth and slight regression of bevacizumab-treated tumors translated to a TGI value of 103.5% (tumor growth inhibition, %TGI = [Tumor Volume_Control Day X_–Tumor Volume_Treated Day X_]/[Tumor Volume_Control Day X_–Tumor Volume_Control Day 0_] × 100), values >100% indicate tumor shrinkage/remission as compared to control tumor group. Additionally, we recorded tumor weights and we found a strong correlation of tumor volumes and weights (*R*^2^ = 0.97) (data not shown).

### MRI of the chronic treatment study (Study A)

**Figure 2:**
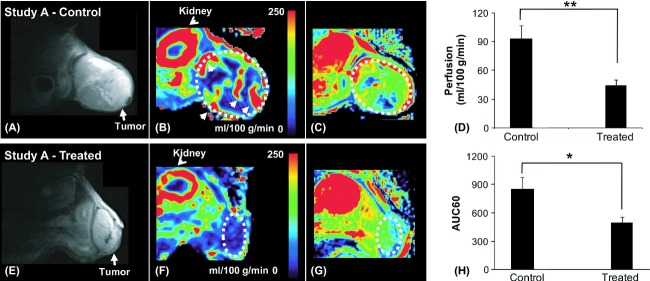
MRI of the chronic treatment group (Study A). *T*_2_-weighted anatomical images (left), corresponding quantified ASL perfusion maps (middle) and DCE AUC60 (right). The treated (E–G) groups show reduction in tumor size and perfusion after bevacizumab treatment compared to the control (A–C). The difference in perfusion as determined by a two-tailed *t*-test is significant with *P*-value of 0.0059 for ASL (D) and 0.0172 for DCE (H). *P < 0.05; **P < 0.01. MRI, magnetic resonance imaging; DCE, dynamic-contrast enhanced; AUC, area under the curve; ASL, arterial spin labeling.

ASL perfusion MRI and DCE-MRI were conducted at day 63 on 10 bevacizumab-treated mice and 10 isotype-control mice. ASL data of one treated mouse were excluded due to large image distortion and DCE data of one control mouse were excluded due to technical issues. In the treated group, along with reduction in size, significant reduction in perfusion was observed (Fig. 2). The mean perfusion in the tumor was 92.6 ± 42.9 (mean±SD) mL/100 g/min in the control group and 44.8 ± 16.1 mL/100 g/min in the treated group, which corresponds to 51.6% difference (*P* < 0.01; two-tailed *t*-test). DCE-MRI also detected significant difference between the two groups (same slice as ASL). The AUC60 was higher in the control mice (852.8 ± 382.0) compared to treated mice (499.5 ± 175.3) (*P* < 0.05).

### Acute treatment study (Study B)

Subsequently, an acute treatment study was designed with the aim to start the imaging procedures at higher tumor volumes with a mean >300 mm^3^ to assess potential acute effects by bevacizumab treatment on hemodynamics in larger, more mature tumor masses. For this study, 30 mice were inoculated with 1.25 million cells, and from day 56 post inoculation onward when any tumor reached the target volume of >200 mm^3^, individual animals were selected for either bevacizumab or isotype control treatment in batches between days 56–88 (for details see Fig. 1B). After 24 h of either treatment, animals were then subjected to MRI scan. Note that the mean tumor volume of all 10 animals selected for acute bevacizumab treatment was 442 and 443 mm^3^ for the isotype control group (range for all animals: 222–640 mm^3^).

As expected, we could not detect any significant decrease in tumor volume within 24 h of bevacizumab treatment; therefore, we additionally selected a small set of six control animals to demonstrate the efficacy of bevacizumab treatment for larger tumor volumes within this experiment (Fig. 1B, after day 85). Even with three animals per group, we could see regression in tumors with a mean volume 270–70 mm^3^ following 20 days of bevacizumab treatment (TGI: 276%).

### MRI of the acute treatment study (Study B)

**Figure 3:**
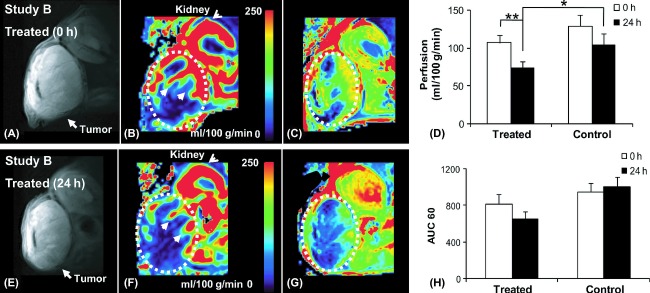
MRI of the acute treatment group (Study B). *T*_2_-weighted anatomical images (left), the corresponding quantified ASL perfusion maps (middle) and DCE AUC60 maps (right). The treated groups did not show change in tumor size but significant reduction before (A–C) and 24 h after (E–G) treatment in perfusion (*P* = 0.006), as well as compared to the control group (*P* = 0.04) as seen in (D). No significant change was found in AUC60 (H). The significance was determined by two-way ANOVA. *P < 0.05; **P < 0.01. MRI, magnetic resonance imaging; DCE, dynamic-contrast enhanced; AUC, area under the curve; ASL, arterial spin labeling; ANOVA, analysis of variance.

In the acute treatment study, the mice were imaged before (0 h) and 24 h after the treatments. ASL data of one mouse were excluded from the control group due to inferior image quality. DCE data of two mice were excluded from the treated group, due to technical issues. At 24 h post dosing, a significant reduction in perfusion was observed in the treated group, but not in the control group (Fig. 3). The tumor perfusion in the treated group reduced from 107.2 ± 32.7 (0 h) to 73.7 ± 27.8 (24 h) mL/100 g/min (*P* < 0.01; two-way ANOVA), which corresponds to 31.0% reduction. The perfusion in the treated mice at 24 h post dosing was also significantly lower than that of the control (*P* < 0.05; two-way ANOVA), which corresponds to 28.8% change. No perfusion change was observed in the control group (129.1 ± 47.5 [0 h] vs 103.6 ± 47.7 [24 h] mL/100 g/min). The AUC60, as measured by DCE, was also reduced in the treated group (656.1 ± 244.4) compared to the control group (999.4 ± 344.5; *P* = 0.06; two-way ANOVA) post dosing, but was not significant compared to predosing (812.6 ± 329.4; *P* = 0.49; two-way ANOVA) (Fig. 3).

### Comparison between ASL and DCE

Operator independent J-index was used to determine the degree of correlation between the perfusion map and the corresponding FLASH image. For Study B, the average J-index pretreatment as measured by the first operator was 0.757 ± 0.096, and as measured by the second operator was 0.754 ± 0.081. Post treatment, the respective values were 0.729 ± 0.099 and 0.725 ± 0.095. As the J-index varies from 0 to 1, with one indicating complete overlap, a value greater than 0.6 was indicative of a strong degree of overlap.

**Figure 4:**
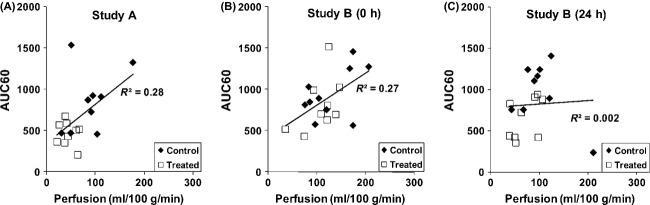
Correlation between ASL and DCE MRI. Correlation between the perfusion and AUC60 from both control and treated animals from the chronic treatment study (Study A) (A), acute treatment study (Study B) at 0 h (B) and at 24 h (C). Correlation was observed between the perfusion and the AUC60 for chronic (*r* = 0.53, *P* = 0.02) and acute treatment at 0 h (*r* = 0.52, *P* = 0.02). The correlation was not significant at 24 h. MRI, magnetic resonance imaging; DCE, dynamic-contrast enhanced; AUC, area under the curve; ASL, arterial spin labeling.

In Study A, correlation (*r* = 0.53, *P* < 0.05) was observed between the perfusion and AUC60 in the control and treated groups (Fig. 4A). In Study B, correlation (*r* = 0.52, *P* < 0.05) was also found between perfusion and AUC60 at 0 h (Fig. 4B). However, no correlation was found at 24 h (Fig. 4C).

### Histological analysis

**Figure 5:**
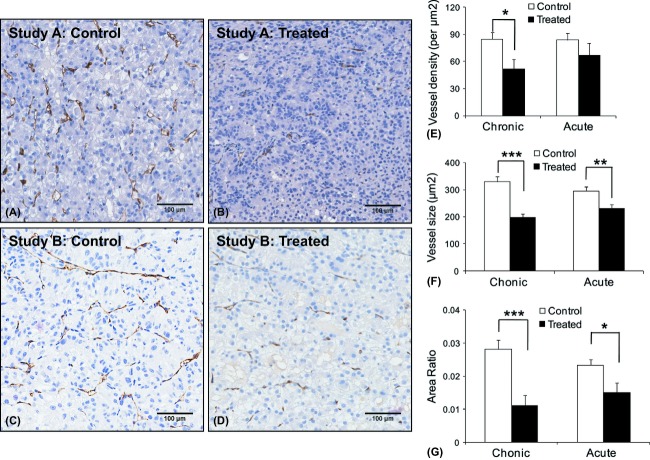
CD34 immunohistochemical staining. Representative CD34 staining of the blood vessels of tumors from control (A) and treated (B) mice in Study A, and control (C) and treated (D) mice in Study B. The calculated vessel density (E), vessel size (F), and area ratio (G) from the CD34 stain of the whole tumor shows significant change after both chronic and acute treatment. After chronic treatment (Study A), significant reduction were observed in the vessel density (*P* = 0.016), vessel size (*P* < 0.001), and area ratio (*P* < 0.001). After 24 h treatment (Study B), only the vessel size (*P* < 0.01), and area ratio (*P* = 0.018) showed significant decrease. The significance was determined by two-tailed *t*-test. *P < 0.05; **P < 0.01; ***P < 0.001.

To evaluate the vascular change, we used the CD34 staining (Fig. 5A–D) to measure the number of vessels per unit tissue area (vessel density), the mean vessel area per vessel (vessel size), and the mean vessel area per unit tissue area (area ratio). In the chronic treatment study, the vessel density (*P* < 0.05; two-tailed *t*-test), vessel size (*P* < 0.01), and area ratio (*P* < 0.001) were significantly reduced in the treated group compared to the control group (Fig. 5E–G). However, in the acute treatment study, while the vessel size showed significant reduction in the treated group compared to the control group (*P* < 0.005), no change of vessel density was observed.

**Figure 6:**
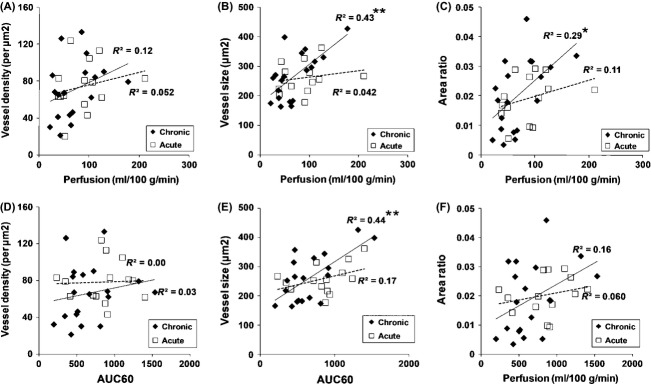
Correlation between MRI measurement and histology. The correlations between perfusion and vessel density (A), vessel size (B), and area ratio (C) in the chronic (solid diamond and line) and the acute (open box and dotted line) studies demonstrate significance between perfusion and vessel size (*r* = 0.66, *P* = 0.0023) and area ratio (*r* = 0.54, *P* = 0.017) in the chronic study (Study A). The correlations between AUC60 and vessel density (D), vessel size (E), and area ratio (F) in the chronic (solid diamond and line) and acute (open box and dotted line) studies show significance between AUC60 and vessel size (*r* = 0.65, *P* = 0.0019) after chronic treatment (Study A). No correlation with histology was observed either for perfusion or AUC60 after 24 h treatment (Study B). *P < 0.05; **P < 0.01. MRI, magnetic resonance imaging; AUC, area under the curve.

Comparing both MRI measurements with histology (Fig. 6), correlation was found between ASL perfusion and area ratio (*r* = 0.54; *P* = 0.017) and vessel size (*r* = 0.66; *P* < 0.005) in the chronic treatment study. Correlation was also found between AUC60 and vessel size (*r* = 0.65; *P* < 0.005) in the chronic treatment study. In the acute treatment study, although perfusion, AUC60, and vessel size were all reduced, no correlations between vessel size and both MRI measures were observed. None of the MRI measures correlated with the vessel density.

## Discussion

The majority of preclinical MRI studies that investigated angiogenesis in tumors outside the brain have been performed using DCE-MRI due to the large signal change and signal-to-noise ratio. To address the quantification issues of DCE-MRI, quantitative tumor perfusion measured by ASL MRI was used to evaluate antiangiogenic treatment response in a mouse xenograft model. Our results show that ASL can detect treatment response to bevacizumab in 24 h in the same animals (the treated group in Study B), while DCE can only detect treatment effect when compared to a control group. This indicates that, besides being noninvasive, the quantitative nature of ASL may be an important factor in this longitudinal study.

This study differs from previous studies in that both acute (24 h after treatment) and chronic (39 days after treatment) effects of bevacizumab were examined and compared in the same mouse model under similar experimental conditions using both ASL and DCE-MRI and subsequently compared with histology. Significant decrease in tumor perfusion after treatment was observed in both studies (summarized in Table 1). Notably, perfusion was found to be reduced even at 24 h after treatment when there was no change in tumor volume. Tumor perfusion correlated with vessel size and vessel area ratio measured from CD34 staining. The reduction in vessel density, size, and area ratio with acute treatment was half of that with chronic treatment (Table 1). Interestingly, the same trend was observed with perfusion as well. This suggests that perfusion may be an early biomarker that reflects change in tumor vasculature.

**Table 1:** Percentage change in treatment versus control for both chronic and acute treatments.

% Change (treated/control)	Chronic treatment study	Acute treatment study (24 h)
ASL perfusion	−51.6	−28.8
DCE AUC60	−41.4	−*34.3*
Tumor volume	−87.2	n/a
Vessel density	−38.8	−*20.3*
Vessel size	−40.2	−21.9
Area ratio	−60.4	−35.8

Italics represent no significance. ASL, arterial spin labeling; DCE, dynamic-contrast enhanced; AUC, area under the curve.

Our results indicate that bevacizumab significantly reduced tumor perfusion at both acute and chronic dosing. Chronic treatment of bevacizumab significantly decreased tumor volume, vessel size (blood volume), and vessel density, while acute treatment only reduced vessel size. Notably, a correlation was found between perfusion and vessel size in the chronic treatment group, but not in the acute treatment group. Tumor perfusion is an indicator of nutrition supply to tumor and has been associated with tumor neovascularization, metabolic demand, and tumor oxygenation. However, changes in vascularity may not necessarily correspond to similar changes in perfusion or vice versa. For example, while both vessel density and vessel size/area were reduced in the chronic treatment, which may implicate suppression of vessel growth and functioning vessels, tumor perfusion only correlated with changes in size/area, not density. On the other hand, in the acute treatment study, the vessel density was not expected to be altered within 24 h and this was confirmed by histology (Fig. 5E). The reduction in perfusion could be attributed to the reduced vessel size/area in this case. Indeed, VEGF inhibitors can cause vessel constriction and hypertension via suppression of nitric oxide production 48. This could lead to reduced perfusion and vessel size/area that was observed in the acute phase.

Perfusion, as measured with ASL has been shown to have a strong correlation with DCE-MRI parameters, such as K_ep_, percent relative enhancement, and percent enhancement ratio in mice tumor models 32. In this study, we compared perfusion of ASL with AUC of DCE-MRI. AUC was selected over other quantitative parameters due to the better reproducibility. A couple of clinical studies have evaluated the semi-quantitative and quantitative DCE analysis in tumors. It was found that semi-quantitative parameters like AUC is highly reproducible, while quantitative parameters like *K*^trans^, exhibited greater variability, so that more individuals are required for the study to be statistically significant 49,50.

We found positive correlation between ASL perfusion and DCE AUC60 in chronic (*r* = 0.53, *P* < 0.05) and at 0 h in acute (*r* = 0.52, *P* < 0.05) treatment studies. However, both parameters measure different aspects of the tumor vasculature: ASL specifically measures blood flow, while AUC represents the accumulation of gadolinium contrast in tissue space over a period. AUC includes a mixing contribution from blood flow, blood volume, vessel permeability to the contrast agent, and the fraction of interstitial space. The lack of correlation between perfusion and AUC60 at 24 h after acute treatment suggests that all these parameters may have been changed by bevacizumab treatment. The loss of correlation may be due to these additional changes other than perfusion. Besides, individual difference in response to treatment may also contribute to the variability. Therefore, more studies will be needed to interpret the underlying change in tumor vasculature from the results of DCE-MRI.

DCE-MRI has been widely used for in vivo measurement of vascular changes. For example, it has been shown that vessel permeability estimated from DCE-MRI correlates with histologic quantification of vascular density as well as with the molecular expression of angiogenic factors in a murine glioblastoma model 51. Furthermore, correlation between AUC and MVD in patients with cervical carcinoma has been reported 52. In this study, we found good correlation between AUC60 and vessel size in the both chronic and acute treatment studies, though there was no correlation with vessel density.

The earliest response (4–8 h) to treatment that has been reported is not of perfusion, but of vascular response measured as R2* changes induced by iron oxide nanoparticles in a thrombogenic vascular targeting agent on fibrosarcoma in mice 53. However, vascular changes which were observed as early as 4–8 h regressed to baseline 24 h after treatment 53. Another study on vascular disrupting agents has contradictory results though, with a marked decrease in T1 enhancement using gadofovest trisodium indicating reduction in blood flow 24 h after treatment 54. The fact that significant reduction in perfusion was observed before any detectable change in tumor volume suggests that perfusion imaging is sensitive to changes in perfusion due to very early response to antiangiogenic treatment. In this study, the bevacizumab treatment successfully inhibited the vessel growth of the A498 tumor cells, which is reflected by suppression in tumor volume either started as early as post inoculation day 23 (Fig. 1A) or as late as day 85 (Fig. 1B). In either case, tumor volume was significantly different at least 5* *days after treatment. However, MRI can detect perfusion changes as early as 24 h after the start of treatment.

There are limitations in this study. First, the estimated tumor perfusion in ASL was based on assuming a constant value of tissue/water partition coefficient, *λ*. However, this value could be very different in tumor 55 and may even change regionally and temporally with tumor progression. A study in a brain tumor model estimated that ∼10% change may be introduced by varying *λ* from 0.9 to one in the brain 31, but no study has reported the value in xenograft tumor. To further improve the accuracy of perfusion quantification in tumor, a measurement of *λ* will be needed 56. Second, the ASL acquisition was obtained from a single slice but not the whole tumor. This scan which took 24 min was based on an imaging protocol optimized for measuring low perfusion accurately as mentioned earlier. This protocol was preferred over faster methods because tumor perfusion is known to be low with high heterogeneity. Covering the whole tumor using multislice EPI can be achieved in the same scan time, but the accuracy will need to be evaluated as multislice acquisition may reduce the perfusion sensitivity and increase variation in arterial transit time due to the need for a larger inversion slab in FAIR ASL. Therefore, 3D volumetric acquisition or other labeling strategies would be desirable in future studies. Third, the 5-*μ*m thick slices acquired for histology were much thinner than the MRI slices and hence the result may not be that representative of the volume that MRI covered and may contribute to the lack of correlation with the MRI results in Figure 6. Changes in animal positioning and the distortion caused by histology preparation could also affect the choice of matching location for comparison. Fourth, while correlating vessel density and perfusion, we did not distinguish between nonfunctional and functional blood vessels, which might be a more accurate indicator of perfusion. Future studies could incorporate a more detailed histological analysis to examine this. Fifth, in our studies, a subcutaneous tumor model was chosen because of the ease of tumor inoculation, imaging and subsequent perfusion quantification. As the surrounding tumor environment can alter the biology of the tumor, further studies on spontaneous or orthotopically grown tumors could predict the response more accurately 6. The methodology that we established can be extended to orthotopic tumors. Finally, no significant change was found in AUC60 at 24 h after the treatment. This may be partly due to the sensitivity of the MRI sequence used. It was noted that the optimal flip angle for *T*_1_-dependent contrast is different from the Ernst angle 57 and its relevance to DCE-MRI was suggested by Evelhoch 58. Considering a tissue *T*_1_ of about 1500 msec at 7T, the optimal flip angle for DCE-MRI would be around 8°. For the flip angle used in this study, this may lead to up to 20% difference in signal change at high Gd concentration.

In conclusion, we demonstrated that quantitative perfusion measured by ASL MRI could reflect changes in tumor vasculature, at early and late phases of the antiangiogenic treatment. It may be used as a quantitative biomarker for prognosis of antiangiogenic treatments. The noninvasive and quantitative nature of this method allows repeated and longitudinal measurements and will enable easy translation from animal models to clinical studies.
